# Intergroup Contact Effects via Ingroup Distancing among Majority and Minority Groups: Moderation by Social Dominance Orientation

**DOI:** 10.1371/journal.pone.0146895

**Published:** 2016-01-11

**Authors:** Mathias Kauff, Katharina Schmid, Simon Lolliot, Ananthi Al Ramiah, Miles Hewstone

**Affiliations:** 1 Institut für Psychologie, FernUniversität Hagen, Hagen, Germany; 2 ESADE Business School, Ramon Llull University, Barcelona, Spain; 3 Department of Psychology, University of British Columbia, Vancouver, British Columbia, Canada; 4 Independent researcher, Kuala Lumpur, Malaysia; 5 Department of Experimental Psychology, University of Oxford, Oxford, United Kingdom; University of Bristol, UNITED KINGDOM

## Abstract

Five studies tested whether intergroup contact reduces negative outgroup attitudes through a process of ingroup distancing. Based on the deprovincialization hypothesis and Social Dominance Theory, we hypothesized that the indirect effect of cross-group friendship on outgroup attitudes via reduced ingroup identification is moderated by individuals’ Social Dominance Orientation (SDO), and occurs only for members of high status majority groups. We tested these predictions in three different intergroup contexts, involving conflictual relations between social groups in Germany (Study 1; *N* = 150; longitudinal Study 2: *N* = 753), Northern Ireland (Study 3: *N* = 160; Study 4: *N* = 1,948), and England (Study 5; *N* = 594). Cross-group friendship was associated with reduced ingroup identification and the link between reduced ingroup identification and improved outgroup attitudes was moderated by SDO (the indirect effect of cross-group friendship on outgroup attitudes via reduced ingroup only occurred for individuals scoring high, but not low, in SDO). Although there was a consistent moderating effect of SDO in high-status majority groups (Studies 1–5), but not low-status minority groups (Studies 3, 4, and 5), the interaction by SDO was not reliably stronger in high- than low-status groups. Findings are discussed in terms of better understanding deprovincialization effects of contact.

## Introduction

“The most dangerous worldview is the worldview of those who have not viewed the world.” (Alexander von Humboldt, n.d.)

“Those with outgroup friends gain distance from their own group and form a less provincial perspective on other groups.” (Thomas F. Pettigrew, 1997)

Both these quotations speak to the notion that intergroup experiences with diverse others can improve attitudes towards members of groups different from one’s own group. This idea is the central tenet of intergroup contact theory [1,2]. In addition, Pettigrew’s statement suggests that getting to know others from different backgrounds may loosen individuals’ bonds to their own group and lead to a less ethnocentric perception of outgroups. Pettigrew’s ‘deprovincialization’ hypothesis proposes that cross-group contact, especially close contact with outgroup members, promotes the humanization of outgroup members and a reappraisal of the importance of ingroup norms and customs [1,3]. Pettigrew [1] supposes that by getting to know outgroup members’ different ways of life, customs, and norms people start realizing that besides ingroup norms and customs there are other ways to evaluate the social world. As a consequence cross-group contact should lead individuals to distance themselves from their ingroup.

In line with the deprovincialization hypothesis, a small, but growing body of research has tested the idea that ingroup distancing is a mediator in the well-established link between contact and outgroup attitudes [4]. The empirical evidence base, however, shows only mixed support for this assumption to date [5–7]. We argue that one possible reason for these ambiguous findings concerns the equivocal link between ingroup identification and outgroup attitudes [8] In the present paper we therefore predict that the indirect effect of contact on outgroup attitudes via reduced ingroup identification is conditional, such that Social Dominance Orientation (SDO; [9]), a preference for hierarchical over egalitarian relations between groups, moderates the deprovincialization effect. Specifically, we predict that reduced ingroup identification is more strongly related to positive outgroup attitudes for individuals high in SDO. Consequently, we expect the indirect effect of contact on outgroup attitudes via reduced identification to occur primarily for individuals high in SDO.

However, based on Social Dominance Theory [9], as we detail below, our predictions should primarily apply for high-status majority groups but not for lower-status subordinate groups. We test these predictions in five studies in different contexts (Germany, Northern Ireland, UK), among majority (Studies 1–5) as well as minority (Studies 3, 4, and 5) groups.

### Intergroup Contact, Ingroup Identification, and Outgroup Attitudes

Intergroup contact theory claims that cross-group contact can improve the quality of intergroup relations because negative attitudes towards outgroups are reduced after getting to know outgroup members [2]. This assumption is supported by a plethora of studies [10]. Particularly impressive, however, are meta-analytic findings [4] indicating, on the basis of 515 studies, that the mean effect size of cross-group contact on intergroup prejudice is *r* = -.22. A special high-quality, powerful form of intergroup contact, which we focus on in this paper, is that of cross-group friendship [11].

Besides showing such primary contact effects (whereby intergroup contact with a given outgroup improves attitudes towards the same outgroup), a number of studies now also provide evidence for more generalized contact effects, so called secondary transfer effects [1,6,12]. Secondary transfer effects involve the extension of prejudice-decreasing contact effects beyond the encountered outgroup to unrelated outgroups [5]. For example, Pettigrew [1] found that among majority group members in France, Germany, Great Britain, and the Netherlands, cross-group friendship with minority group members was associated with increased positive attitudes not only towards the encountered minority groups, but also towards secondary outgroups not present in each of the countries studied.

Based on these findings, Pettigrew [5] argued that cross-group contact (especially in the form of cross-group friendship) may lead individuals to adopt a deprovincialized perspective on the social world, involving a distancing from, and reappraisal of, the ingroup, which may then explain positive effects on outgroup attitudes, including secondary outgroup attitudes. In a more recent investigation, Pettigrew [5] obtained support for the assumption that a process of ingroup distancing underlies contact effects. Based on data derived from a German probability sample he showed that positive cross-group contact with immigrants was associated with reduced national identification, which then decreased negative attitudes towards homosexuals. However, not all studies show such clear-cut results. Tausch et al. [6] found only limited evidence for a mediating role of ingroup distancing. While in their first study ingroup reappraisal mediated the effect of contact with the outgroup in Cyprus (i.e., Greek or Turkish Cypriots) on attitudes towards Greeks and Turks in general, the authors failed to replicate this finding in three further studies in Northern Ireland and the USA [13]. Eller and Abrams [14] also reported that reduced national identification did not mediate the effect of contact on outgroup attitudes in the context of intergroup relations between English and French people as well as between Americans and Mexicans.

It is, perhaps, not surprising that studies testing the mediating role of ingroup distancing in the relationship between cross-group contact and outgroup attitudes have received mixed support to date. First, the influence of ingroup distancing has often been researched in studies with a focus on attitudes towards secondary outgroups [6,13]. The influence of cross-group contact on attitudes towards primary outgroups via ingroup distancing has seldom been investigated. In one rare example, Verkuyten et al. [7] demonstrated in three different Dutch adolescent samples that cross-group contact not only influenced ingroup distancing (operationalized as reduced ingroup identification and positive feelings towards the ingroup) but also attitudes towards multiculturalism (Studies 1–3) and feelings of intergroup threat (Study 3). However, Verkuyten et al. [7] focused only on the effects of contact on ingroup distancing and did not extend these findings to attitudes towards particular outgroups. Although attitudes towards multiculturalism and intergroup threat have been shown to be highly related to outgroup attitudes [15,16], we consider it crucial to directly study the relationship between contact, ingroup distancing and attitudes towards primary outgroups. It is of great importance to understand the link between ingroup distancing and primary outgroup attitudes before drawing wider conclusions about the involvement of ingroup distancing in understanding attitudes toward secondary outgroups.

Second, ingroup distancing has often been operationalized as a reduction of ingroup identification or positive attitudes towards the ingroup, yet it has long been known that ingroup attitudes typically tend to be inconsistently related to outgroup attitudes [17,18,19]. Indeed, Brewer [8] has called into question the idea that ingroup and outgroup evaluation are inversely related. She suggests that a negative relationship between ingroup identification and outgroup attitudes is more likely when the intergroup context is characterized by competition between groups [20,21,22].

Based on this notion, we argue that the relationship between ingroup identification and (primary) outgroup attitudes is conditional. Specifically, we predict that the relationship is qualified by SDO, a preference for hierarchical intergroup relations that is related to a perception of the world as a dangerous and competitive place [23,9]. Consequently, we expect that the effect of ingroup distancing on outgroup attitudes occurs only for individuals high in SDO. Thus, the indirect effect of cross-group friendship on outgroup attitudes via ingroup distancing should be found for individuals with relatively high scores in SDO but not for those with low SDO scores.

As we outline in more detail below, we additionally suggest that the ingroup’s status is of importance when considering the mediating role of ingroup distancing; we expect that the effect of ingroup distancing on outgroup attitudes among individuals high in SDO will occur for high-status majority groups only.

### SDO, Ingroup Identification, and Outgroup Attitudes

Social Dominance Theory posits that individuals can differ with regard to their endorsement of group-hierarchies, that is their Social Dominance Orientation [24]. Going beyond this original definition of SDO, Duckitt [25] describes SDO as a competition-driven motivation for ingroup dominance and power over other groups. Hence, SDO in Duckitt’s sense needs to be considered as an ingroup-based phenomenon: Individuals with high SDO make strategic use of hierarchy-enhancing ideologies, such as prejudice, to establish or maintain the superiority of the ingroup [9]. In line with this notion, numerous studies indicate that SDO is negatively related to outgroup attitudes [26,27]. In addition, SDO and ingroup identification seem to interact when predicting outgroup attitudes. Perry and Sibley [28], for example, showed that the relationship between SDO and racism was stronger when ethnic identity (vs. personal identity) was salient. Also, Sidanius, Liu, Shaw, and Pratto [29] found the strongest ingroup favoritism for High-SDO individuals who were highly identified with their ingroup [30].

Based on this evidence, we expect that the link between ingroup identification and outgroup attitudes is especially strong for individuals high in SDO [31]. We assume that reduced ingroup identification following from cross-group friendship should decrease the necessity of strategic outgroup devaluation for High-SDO individuals [32]. We thus predict that High-SDO individuals whose identification with their ingroup decreases as a consequence of cross-group friendship should be less motivated to devalue outgroups in a particular intergroup context, despite having a general need for hierarchy. As a consequence, an effect of cross-group friendship on outgroup attitudes via ingroup distancing (operationalized as reduced ingroup identification) should occur primarily for High-SDO individuals. Due to reduced ingroup identification through cross-group contact, High-SDOs should be less likely to use hierarchy-enhancing strategies, such as outgroup devaluation, to maintain the status of their ingroup [9,33].

By studying the moderating effect of SDO on the indirect effect of cross-group friendship on outgroup attitudes via ingroup identification, we extend previous research on the moderating role of SDO in the relationship between contact and outgroup attitudes. Studies in this area have so far yielded ambiguous results. In a study with British prison inmates, [34] showed that effects of contact on attitudes toward members of ethnic outgroups were especially strong for High-SDO individuals. Dhont and Van Hiel [35] were able to replicate this finding in a more heterogeneous sample. Contrary to these findings, results from a study with a cross-European representative sample carried out by Schmid, Hewstone, Küpper, Zick, and Wagner [12], indicate that “the effectiveness of contact may in fact be limited for individuals who believe strongly in group-based hierarchies, namely, who are high in social dominance orientation” (p. 32)–that is in this study contact was especially effective for participants *low* in SDO. In line with this, Asbrock, Christ, Duckitt and Sibley [36] demonstrated that contact reduced anti-immigrant attitudes only for Low-SDO individuals but not for High-SDO individuals in a German population sample. Asbrock et al. [36] argue that High-SDOs should be most reluctant to change their attitudes because of the instrumentality of prejudice for the maintenance of ingroup-superiority. However, neither Asbrock et al. [36] nor Schmid et al. [12] took the underlying process of dis-identification as a consequence of cross-group friendship into account while studying the moderating role of SDO in the link between contact and outgroup attitudes. As outlined above, we argue that reduced identification should decrease the instrumentality of hierarchy-enhancing strategies, such as prejudice. Hence, the relationship between cross-group friendship and outgroup attitudes via reduced identification should be especially strong for High-SDO individuals. More precisely, we predict that a reduction of ingroup identification as a consequence of cross-group friendship is related to positive outgroup attitudes for individuals high in SDO but not for those low in SDO.

Asbrock et al. [36] additionally suggest that moderation effects of SDO are context-dependent [37]. In our study, we consider this notion in two ways: First, we expect–as outlined in the next section–that SDO will moderate the relationship between ingroup identification and outgroup attitudes only for high-status groups. Second, we test our predictions across a variety of contexts (i.e., intergroup relations between Germans and immigrants in Germany, Protestants and Catholics in Northern Ireland, as well as between White British and ethnic minorities in the UK).

### The Influence of Ingroup Status

The aforementioned predictions should hold primarily for high-status majority groups, but not for low status minority groups, for two reasons. First, SDO differentially affects evaluations of the ingroup and of outgroups in high- vs. low-status groups. For example, SDO predicts pro-ingroup behavior only in high-status groups but not in low-status groups [38]. Moreover, High-SDOs are prejudiced against groups that are perceived as legitimately subordinate (i.e. low-powered minority groups [25]). Therefore, confrontation with low-status minority groups should activate outgroup devaluation in high-status groups members high in SDO. For individuals high in SDO from low-status groups who are high in SDO, however, (superiority-motivated) outgroup devaluation should not be activated by confrontation with high-status groups [39]. Research shows that within stable status hierarchies, low-status individuals high in SDO might show outgroup devaluation with regard to other low-status groups but should also favor high-status (out)groups [40]. Henry, Sidanius, Levin, and Pratto [41], for example, demonstrated that in a Lebanese sample (considered to be a low-status group in comparison to the USA) SDO was negatively related to aggression against the outgroup (USA) while in the high-status US-sample a positive correlation between SDO and aggression against the Middle East occurred. Moreover, and in line with these results, low-status group members who are high in SDO tend to be low in ingroup identification [19].

Second, ingroup distancing is more likely to apply for majority than for minority group members. Ingroup distancing (e.g., re-evaluation of ingroup norms and traditions) through contact seems to be more likely for those who have not had much experience of divergent ways of life prior to the contact experience. In accordance with this reasoning, Al Ramiah, Hewstone, Voci, Cairns, and Hughes [42] showed that the effect of contact on prejudice is greater for those who have had less intergroup contact in the past. However, members of minority groups are typically more exposed to, and accustomed to dealing with, different norms and traditions. For example, minority individuals often engage in processes of cultural frame-switching [43], and may integrate two or more cultural identities into their sense of self [44]. Because minority individuals may thus be more adept at negotiating their various identities, intergroup contact with the majority group should be less likely to lead to processes of ingroup distancing.

In line with this reasoning, research on dual identities [45] points out that members of low-status minority groups prefer maintenance of their ingroup identity when nested in a superordinate group over a rejection of their identity in the course of recategorization in a common group composed of minority and majority members [46,47].

### The Present Research

**Fig 1:**
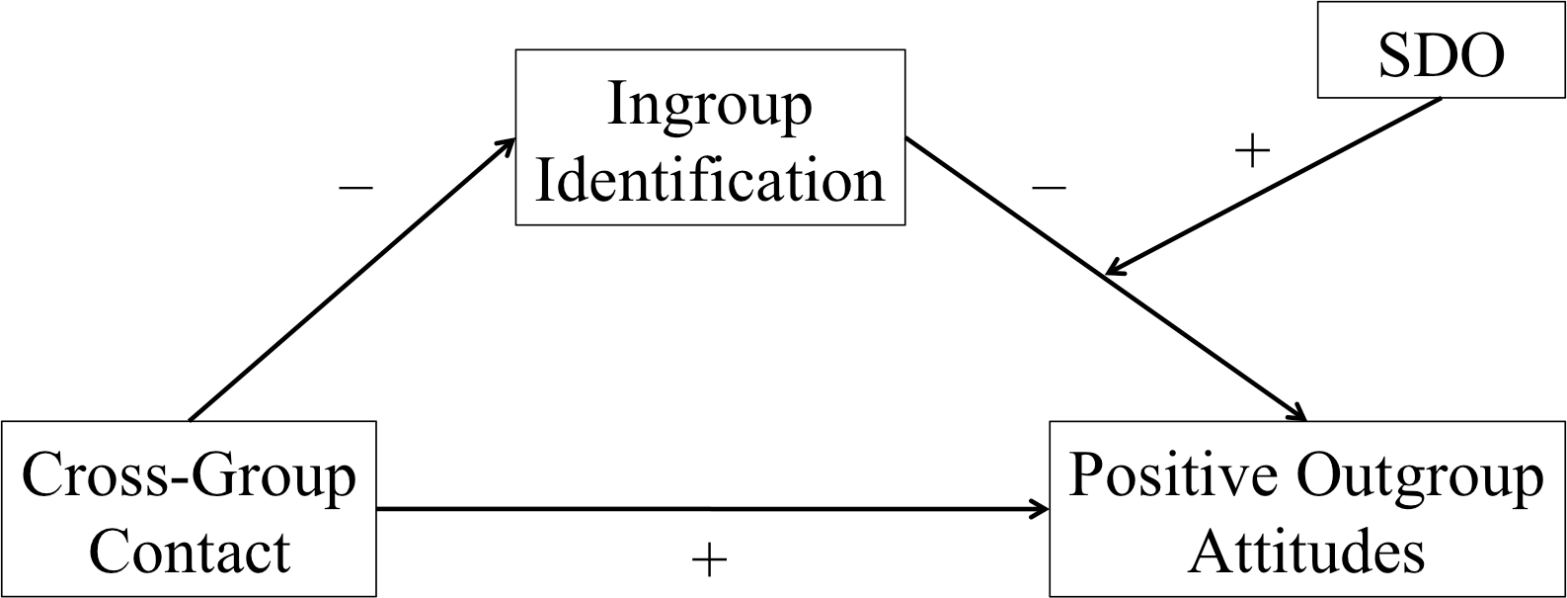
Theoretical model to be tested in Studies 1–5.

The aim of the present research is to further examine the notion that cross-group friendship improves outgroup attitudes through a process of ingroup-distancing and thus to extend the existing evidence base in important ways. Drawing on prior research on deprovincialization [5,7], as well as on Social Dominance Theory [19,28,48] we expect that the indirect effect of cross-group friendship on outgroup attitudes via ingroup identification is qualified by individuals’ SDO. In so doing, our research seeks to shed light on the conditions under which a deprovincialization effect occurs, a previously untested moderation hypothesis, which may explain the mixed evidence base on ingroup-distancing to date. More precisely, we predict that the relationship between reduced identification and negative outgroup attitudes is moderated by SDO, such that a significant negative relationship between identification and outgroup attitudes will be obtained for individuals who are high, but not low in SDO. We therefore argue that reduced ingroup identification as a consequence of cross-group friendship translates into more positive outgroup attitudes only for individuals with high SDO (see Fig 1 for an overview of our theoretical model).

Moreover, we additionally hypothesize that this indirect effect of cross-group contact on outgroup attitudes via reduced ingroup identification occurs primarily for high-status, but not low-status groups because we do not expect cross-group contact to prompt dis-identification among minority groups to the same extent as among majority groups. We also do not expect the interaction between ingroup identification and SDO to occur for low-status minority groups.

We tested these hypotheses in five independent samples. In Study 1, we tested our hypothesized moderated mediation model among non-immigrant German undergraduate students. In Study 2 we utilized a longitudinal German probability sample to replicate findings of Study 1 over time. While Studies 1 and 2 focused on the high-status majority perspective only, Studies 3–5 additionally considered the perspective of low status minority groups: In Studies 3 and 4, we used survey data from Catholic and Protestant adults in Northern Ireland. In Study 5, we tested our predictions in the context of intergroup relations between White and Asian British students in an ethnically mixed British high school. Finally, we present a quantitative summary of our findings by conducting a meta-analysis across our five studies.

## Study 1

Study 1 examined whether the relationship between cross-group friendship and positive attitudes towards outgroup members is mediated by reduced ingroup identification. Moreover, we studied whether the relationship between identification and outgroup attitudes is especially strong for individuals high in SDO. Study 1 was conducted in the context of immigration in Germany: non-immigrant participants were asked about their contact experiences as well as their attitudes towards immigrants.

### Method

#### Participants and Procedure

Data were collected at a German university. Participants were undergraduate psychology students. Data collection of Study 1 was part of an introductory course in psychology. Due to the regularity of such surveys and the approval of several identical studies by the local ethics committee at the psychology department at Philipps-University Marburg this particular study did not pose enough of a serious ethics risk to obtain formal ethics approval. Also, no formal ethics waiver was requested. Participants were assured that participation is voluntary, can be cancelled at any time and that data were anonymized. Informed consent was provided in written format. No minors were surveyed. Therefore no written informed consent from parents or guardians needed to be requested. The data file is available in the Open Science Framework: https://osf.io/5hkm4/.

The original sample size for Study 1 was *n* = 221 (i.e., all participants that completed the questionnaire). However, we excluded participants with a migration background, those participants that failed to indicate their nationality (*n* = 68), and those who had missing values on all relevant variables (*n* = 3). Of the remaining 150 students, 36 were males and 114 females (*M*_*age*_ = 22.04; age range: 18–44 years). The study was announced at a lecture and the questionnaire was provided online in August 2011.

#### Measures

Among a number of measures unrelated to the present study, participants completed measures of cross-group friendship, national identification, attitudes towards immigrants, and SDO. Unless otherwise stated all items had response scales ranging from 1 = *complete disagreement* to 7 = *full agreement*. *Cross-group friendship* was measured with a single item (‘How many of your friends are immigrants?’). This item was measured on a scale ranging from 1 = *none* to 7 = *many*. *Identification* was measured with three items (‘Being German is an important part of my personality’, ‘I identify with Germans as a group, ‘I am proud to be German’; α = .88). *Attitudes towards immigrants* was also measured with three items (‘Most German politicians care too much for immigrants and too little for the average German’, ‘There are too many immigrants living in Germany’, ‘Immigrants living in Germany should be sent back to their home country, if jobs become scarce’; α = .88; items were recoded so that higher scores represent more positive attitudes). SDO was measured with a German 15-item scale (e.g., ‘Inferior groups should stay in their place’, ‘Some groups of people are simply inferior to other groups’; α = .89; [49]). The scale is based on the SDO scale by Pratto et al. [24].

### Results and Discussion

We first tested the direct effect of cross-group friendship on attitudes toward immigrants without considering the indirect effect via identification. We then proceeded to test the full model. This model involved the indirect and conditional indirect effects of cross-group friendship on attitudes towards immigrants via ingroup identification, and the possible moderating role of SDO. We tested a moderated mediation model in which SDO moderated the path between ingroup identification and attitudes towards immigrants. We used bootstrapping procedures as recommended by Preacher and Hayes [50]. To test indirect effects for significance, we utilized the product-of-coefficients-approach [51]. Ingroup identification and SDO were mean centered prior to analyses to avoid multicollinearity [52]. Table 1 reports means, standard deviations, and correlations of all variables.

**Table 1:** Means, standard deviations, and correlations for variables in Study 1.

Variable	Cross-group friendship	Ingroup identification	Pos. outgroup attitudes	SDO
Cross-group friendship	–	-.33**	.26**	-.20*
Ingroup identification		–	-.31**	.25**
Positive outgroup attitudes			–	.67**
SDO				–
*M*	3.23	3.35	5.78	2.36
*SD*	1.59	1.48	1.17	.82

Note: SDO = Social Dominance Orientation.

*p < .05

**p < .01.

#### Moderated mediation analyses

The direct effect of cross-group friendship on positive outgroup attitudes (without inclusion of ingroup identification) was significant (*b* = .20, *CI*_*95%*_ = .08, .31, *p* = .001). However, in the full moderated mediation model, no significant direct relationship between cross-group friendship and positive outgroup attitudes emerged (*b* = .07, *CI*_*95%*_ = .00, .14, *p* = .12). Instead, cross-group friendship was negatively associated with ingroup identification (*b* = -.30, *CI*_*95%*_ = -.43, -.19, *p* = .001), and identification was associated with less positive outgroup attitudes (*b* = -.10, *CI*_*95%*_ = -.17, -.01, *p* = .02), as was SDO (*b* = -.91, *CI*_*95%*_ = -1.04, -.77, *p* = .001).

We did not find a significant indirect effect of cross-group friendship on positive outgroup attitudes via ingroup identification (*b* = .07, *CI*_*95%*_ = .16, -.03, *p* = .17). However, in line with our predictions, the effect of identification on outgroup attitudes was moderated by SDO (*b* = -.15, *CI*_*95%*_ = -.26, -.04, *p* = .01) resulting in a significant conditional indirect effect for High-SDOs (+1 *SD* above the mean; *b* = .07, *CI*_*95%*_ = .02, .13, *p* = .01) and a non-significant conditional indirect effect for Low-SDOs (-1 *SD* below the mean; *b* = -.01, *CI*_*95%*_ = -.04, .03, *p* = .70).

Please note that we additionally checked for a moderation effect of SDO on the direct effect of cross-group friendship on outgroup attitudes in Study 1. In line with Study 2 by Dhont and Van Hiel [35], SDO did not moderate the relationship between cross-group friendship and outgroup attitudes (*p* = .72). Moreover, we tested an alternative model in which outgroup attitudes are predicted by cross-group friendship, SDO, and identification as well as the interactions between these variables (including the three-way interaction). No effect of cross-group friendship (*p* = .26) or identification (*p* = .052) on outgroup attitudes emerged. However, we found an effect for SDO (*p* < .001). No interactions–besides the interaction between SDO and identification (*p* = .033)–was significant (*p*s > .169).

Study 1 thus supported our predictions, by showing that the indirect effect of cross-group friendship on outgroup attitudes via reduced ingroup identification was qualified by SDO: reduced identification was associated with positive attitudes towards immigrants for participants high in SDO (but not for participants with low SDO scores). However, Study 1 was cross-sectional in nature. In order to ascertain the obtained relationships over time we sought to replicate our findings longitudinally in Study 2.

## Study 2

Data in Study 2 were again drawn from the German context and dealt with Germans’ attitudes towards foreigners (for an analysis of the content of the term foreigner in Germany, see [53]). We used a two-wave longitudinal probability dataset, which allows us to derive conclusions about the relationship between constructs over time and be more confident about the causal order of the constructs under research [54]. Because we were particularly interested in the causal relation between ingroup identification and outgroup attitudes, we sought to predict attitudes towards foreigners measured in the second wave of the study, by cross-group friendship, ingroup identification, SDO and the interaction of SDO and identification (all measured in the first wave of the study), while controlling for attitudes towards foreigners measured in the first wave of the study.

### Method

#### Participants and Procedure

Data were derived from a panel study carried out by a professional survey company (using computer aided telephone interviewing techniques). We followed the strict ethics code of the survey company (name of research company: Infratest Sozialforschung, München). Therefore no formal ethics waiver or approval was requested. Participants were assured that participation is voluntary, can be cancelled at any time and that data were anonymized. Informed consent was provided orally since that was the only practical means of doing so without compromising people's anonymity. Participants’ verbal consent was documented by the research company. The research company did not collect any identifying information such as name, address or telephone number from participants. Phone numbers were created by a computer program and were not visible to interviewers. Participants’ names were not recorded. Therefore data were anonymous when handed over to researchers. The data file is freely available under https://dbk.gesis.org/dbksearch/GDESC2.asp?no=0034&search=GMF.

From the 2000 participants that took part in the first wave of the study (t1), conducted between May and June 2006, 771 individuals also participated in the second wave (t2), conducted between June and July 2008. Please note that although the attrition rate seems to be rather high, it needs to be kept in mind that the first wave of the study was carried out as part of a larger sample survey. Of this sample, only a randomly selected half of the respondents were approached again as potential respondents in subsequent survey waves. While there were no mean difference between participants who took part at t1 and t2 and those who dropped out after t1 for cross-group friendship and ingroup identification (*p*s > .11), there was a significant mean difference for SDO (*T*(1939) = 3.99, *p* < .001, *d* = .17; *M*_*t1*_ = 1.70, *SD*_*t1*_ = .62; *M*_*t2*_ = 1.59, *SD*_*t2*_ = .55) as well as for attitudes towards the outgroup (*t*(1927) = 4.17, *p* < .001, *d* = .19; *M*_*t1*_ = 1.58, *SD*_*t1*_ = .87; *M*_*t2*_ = 1.75, *SD*_*t2*_ = .84). However, and importantly, the pattern of correlations between variables was similar for participants who took part at t1 and t2 and those who dropped out after t1 (for all tests for difference of correlation coefficients: *p*s > .33).

The focus of Study 2 was on Germans’ attitudes towards foreigners. Eighteen participants were excluded because they had no German citizenship. Of the remaining 753 participants 344 were male and 409 female (*M*_*age*_ = 47.06; age range: 16–84 years). Respondents completed a larger battery of items, of which we use only a subsample in the present paper [55].

#### Measures

Among a number of measures unrelated to the present study, participants completed measures of cross-group friendship (with outgroup members), ingroup identification, attitudes towards the outgroup, and SDO. *Cross-group friendship* was measured using one item (‘How many of your friends and acquaintances are foreigners living in Germany?’, 1 *= none*, 4 *= many*). *Ingroup identification* was measured using two items focusing on ingroup pride (‘I am proud to be German’, ‘I am proud of Germany’s history’; 1 = *strong disagreement* to 4 = *full agreement*). The items were positively correlated (*r* = .35, *p* < .001) and treated as a combined index. *Outgroup attitudes* were measured using two items (‘There are too many foreigners living in Germany’, ‘Foreigners living in Germany should be sent back to their home country if jobs become scarce’; 1 = *strong disagreement* to 4 = *full agreement*). The items were positively correlated (t1: *r* = .59, *p* < .001; t2: *r* = .58, *p* < .001) and treated as a combined index. SDO was measured with three items adapted from Pratto et al. [24]; ‘Those groups with a low status in our society should keep their low status’, ‘There are groups in the population who are less worthy than others’, ‘Some groups in the population are more useful than others’). The three items formed a reliable scale (*α* = .73).

### Results and Discussion

As in Study 1, we first tested the direct effect of cross-group friendship (t1) on attitudes toward foreigners (t2) without considering the indirect effect via identification but while controlling for attitudes towards foreigners measured at t1. We then proceeded to test the full model, involving the indirect and conditional indirect effects of cross-group friendship (t1) on attitudes towards immigrants (t2) via ingroup identification (t1) and the hypothesized moderating role of SDO in a moderated mediation model in which SDO (t1) moderated the path between ingroup identification (t1) and outgroup attitudes (t2). As mentioned above, we also included outgroup attitudes (t1) in the full model.

Table 2 reports means, standard deviations, and correlations of all variables.

**Table 2:** Means, standard deviations, and correlations for variables in study 2.

Variable	Cross-group friendship (t1)	Ingroup identification (t1)	SDO (t1)	Pos. outgroup attitudes (t1)	Pos. outgroup attitudes (t2)
Cross-group friendship (t1)	–	-.13**	-.07	.30**	.27**
Ingroup identification (t1)		–	.20**	-.23**	-.21**
SDO (t1)			–	-.43**	-.33**
Pos. outgroup attitudes (t1)				–	.74**
Pos. outgroup attitudes (t2)					–
*M*	1.89	2.48	1.59	2.75	2.86
*SD*	.70	.63	.56	.83	.79

Note: SDO = Social Dominance Orientation.

**p < .01.

#### Moderated mediation analyses

The direct effect of cross-group friendship at t1 on positive outgroup attitudes at t2 (while controlling for outgroup attitudes at t1) was significant (*b* = .07, *CI*_*95%*_ = .02, .13, *p* = .01).

In the full moderated mediation model, outgroup attitudes at t1 was a significant predictor of attitudes at t2 (*b* = .67, *CI*_*95%*_ = .62, .72, *p* < .001). Also, a significant direct relationship between cross-group friendship at t1 and positive outgroup attitudes at t2 emerged (*b* = .07, *CI*_*95%*_ = .02, .12, *p* = .01). Furthermore, cross-group friendship at t1 was negatively associated with ingroup identification at t1 (*b* = -.12, *CI*_*95%*_ = -.05, -.18, *p* < .001). Identification at t1 was unrelated to outgroup attitudes at t2 (*b =* -.05, *CI*_*95%*_ = -.12, .01, *p* = .09), as was SDO at t1 (*b* = -.03, *CI*_*95%*_ = -.10, .05, *p* = .53).

We did not find a significant indirect effect of cross-group friendship at t1 on outgroup attitudes at t2 via ingroup identification at t1 (*b* = .01, *CI*_*95%*_ = .00, .03, *p* = .13). However, the effect of identification at t1 on outgroup attitudes at t2 was moderated by SDO measured at t1 (*b* = -.12, *CI*_*95%*_ = -.22, -.01, *p* = .03) resulting in a significant conditional indirect effect for High-SDOs (+1 *SD* above the mean; *b* = .01, *CI*_*95%*_ = .002, .03, *p* < .05) and a non-significant conditional indirect effect for Low-SDOs (-1 *SD* below the mean; *b* = .00, *CI*_*95%*_ = -.01, .02, *p* = .35). Please note that SDO did not moderate the relationship between cross-group friendship at t1 and outgroup attitudes at t2 while controlling for outgroup attitudes at t1 (*p* = .99) in Study 2. We also tested an alternative model in which outgroup attitudes at t2 were predicted by cross-group friendship, SDO, and identification as well as the interactions between these variables (including the three-way interaction) at t1 (controlling for the influence of outgroup attitudes at t1); this latter model yielded a significant effect of cross-group friendship (*p* = .027) and identification (*p* = .027) on outgroup attitudes. However, we did not find an effect of SDO (*p* = .376). Most importantly, no interactions–besides the interaction between SDO and identification (*p* = .022)–were significant (*p*s > .05).

Please also note that we also used a half-longitudinal design as proposed by Cole and Maxwell [56], where we modeled the effect of cross-group friendship (t1) on ingroup identification (t2) (while controlling for ingroup identification, t1) as well as the effect of ingroup identification (t1), SDO (t1), SDO (t1), and the interaction term (t1) on outgroup attitudes (t2) (while controlling for outgroup attitudes, t1). There was only a small effect of friendship measured at t1 on ingroup identification measured at t2 (*b* = -.09, *CI*_*95%*_ = -.16, -.02, *p* = .006) that was no longer reliable when controlling for outgroup attitudes at t1 (*b* = -.01, *CI*_*95%*_ = -.06, .04, *p* = .70). It is, perhaps, not surprising that we could not replicate the effect of cross-group friendship on identification, given the two-year gap between both measurements.

Study 2 thus supported our predictions, by showing that the indirect effect of cross-group friendship on outgroup attitudes via reduced ingroup identification over time was qualified by SDO: reduced identification at t1 was associated with positive attitudes towards foreigners at t2 for participants high in SDO (but not for participants with low SDO scores). Hence, we were able to replicate the findings from Study 1. The results show a longitudinal relationship between cross-group friendship and ingroup distancing on the one hand and more positive outgroup attitudes on the other hand for individuals high, but not low, in SDO. Despite their success in confirming our predictions, Studies 1 and 2 are limited in two ways: in both studies cross-group friendship was measured with only a single item, and all respondents were majority group members. In Study 3 we aimed to address these limitations and replicate our findings in a more conflictual intergroup context, using a more robust measure of cross-group friendship, and in a sample of majority and minority members.

## Study 3

Study 3 was conducted in the context of intergroup relations in Northern Ireland, a setting that has a long history of conflict between those who want Northern Ireland to remain part of the United Kingdom (predominantly Protestants) and those who want it to be reunited with the Republic of Ireland (predominantly Catholics). Similar to Studies 1 and 2, we examined the relationship between cross-group friendship (with the ethno-religious outgroup), ingroup identification (with the ethno-religious ingroup) and general outgroup attitudes (towards the ethno-religious outgroup), while further probing whether levels of SDO moderated potential indirect effects of cross-group friendship on attitudes via identification. We further sought to examine whether effects were comparable across the two ethno-religious groups. Catholics are most commonly considered the historically disadvantaged, minority status group in this context, while Protestants are considered, historically, to be the majority status group. However we also note that majority versus minority status is no longer so clear-cut in this context. For example, recent population statistics indicate that Protestants only marginally outnumber Catholics in Northern Ireland (45.14% Catholics, 48.36% Protestants; see http://www.nisra.gov.uk/Census/key_report_2011.pdf), which makes Protestants less of a numerical majority than before. Moreover, it has been argued in recent years that Protestants feel increasingly marginalized and alienated in Northern Ireland [57], which may suggest that this group considers their majority status as precarious. Thus, although we refer to Protestants as the majority/high-status group and Catholics as the minority/low-status group for the purpose of this paper (considering their historical status differentials), we ask the reader to keep these potential complexities in mind.

### Method

#### Participants and Procedure

One hundred and sixty students studying at the University of Ulster completed a pen-and-paper questionnaire. The questionnaire was approved by Oxford University's Central University Research Ethics Committee (CUREC). Informed consent was provided in written format. The data file is available in the Open Science Framework: https://osf.io/5hkm4/.

Twenty-five participants were dropped from the analyses because they were not born in Northern Ireland. A further three participants were deleted from the analyses because two put their religious identification as "other" and one failed to indicate what their religious affiliation was. Therefore, the final sample consisted of 132 participants (*M*_age_ = 21.83, age range 18 to 42). The sample consisted of 76 Protestants (23 males and 53 females) and 56 Catholics (23 males and 33 females). Data were collected in September 2010.

#### Measures

Respondents were asked about their contact with and attitudes towards the ethno-religious outgroup, SDO, and various demographic variables. *Cross-group friendship* with the ethno-religious outgroup was ascertained using three questions (‘About how many of your friends in your hometown are from the other religious community’ (1 = *none*, 5 = *all*); ‘How often do you visit them in their home?’; and ‘How often do they visit you in your home?’ (1 = *never*, 5 = *very often*)). These three items formed a reliable scale (*α* = .91). *Ingroup identification* was measured using the three items (‘Being a member of my own religious group is an important part of who I am’; ‘Overall, being a member of my own religious community has very little to do with how I feel about myself’ (R), and ‘I feel strong ties with other members of my own religious community in Northern Ireland’; 1 = *strongly disagree* to 5 = *strongly agree*). The three items (with items marked R reverse scored) formed a reliable scale (*α* = .82). *Outgroup attitudes* were measured using a single item feeling thermometer ranging from 0 = *extremely unfavorable* to 100 = *extremely favorable*: ‘How do you feel about <OUTGROUP>? Please rate <OUTGROUP> on a thermometer that runs from zero to a hundred degrees. The higher the number, the warmer or more favorable you feel towards <OUTGROUP>. The lower the number, the colder or less favorable you feel. If you feel neither warm nor cold towards members from <OUTGROUP>, rate them at 50’. SDO was measured using Pratto et al.’s [24] full 16-item SDO scale (0 = *strongly disagree*, 6 = *strongly* agree). The scale showed good reliability (*α* = .87).

### Results and Discussion

#### Preliminary analyses

We carried out a series of analyses of variance (ANOVAs) to test for group differences between Protestants and Catholics on all variables. Results showed no differences between Protestants and Catholics respondents on any of the variables included in the analyses in this sample (all *F*s (1,130) ≤ 2.69, all *p*s *>* .10, all *η*^*2*^ < .02). In addition, we tested whether our model was structurally invariant for Protestants and Catholics, by comparing a model in which we allowed regression coefficients (for relationships between key theoretical variables) to freely vary across the groups to one in which we constrained regression coefficients to be equal. The results of this test indicated that the model was structurally non-invariant for Protestants and Catholic respondents (Δ*χ*^*2*^ = 12.23, *df* = 4, *p* < .02). We therefore performed the analyses separately for each ethno-religious group. We used the same analysis approach as in Studies 1 and 2. Table 3 reports means, standard deviations, and correlations of all variables.

**Table 3:** Means, standard deviations, and correlations for variables in Study 3 for Catholics (above the diagonal) and Protestants (below the diagonal)

Variable	Cross-group friendship	Ingroup identification	Pos. outgroup attitudes	SDO	*M*_Catholics_	*SD*_Catholics_
Cross-group friendship	–	-.27*	.33*	.05	2.19	1.19
Ingroup identification	-.23*	–	-.04	.22	3.42	1.11
Positive outgroup attitudes	.17	-.33**	–	-.31*	70.18	23.63
SDO	-.28*	.02	-.33**	–	2.41	.77
*M*_Protestants_	2.42	3.11	71.05	2.53	-	-
*SD*_Protestants_	1.03	1.06	20.63	.85	-	-

Note: SDO = Social Dominance Orientation.

*p < .05

**p < .01.

#### Moderated mediation analyses for Protestants

The direct effect of cross-group friendship on positive outgroup attitudes was not significant (*b* = 3.42, *CI*_*95%*_ = -0.94, 8.02, *p* = .12).

In the full moderated mediation model, no significant direct relationship between cross-group friendship with the ethno-religious outgroup and positive outgroup attitudes emerged (*b* = .04, *CI*_*95%*_ = -5.32, 4.25, *p* = .99). However, cross-group friendship was negatively associated with ingroup identification (*b* = -.24, *CI*_*95%*_ = -.49, -.01, *p* = .043) and identification was negatively associated with positive outgroup attitudes (*b* = -7.70, *CI*_*95%*_ = -12.12, -3.75, *p* < .001), as was SDO (*b* = -7.64, *CI*_*95%*_ = -12.57, -1.71, *p* = .003).

The indirect effect of cross-group friendship on outgroup attitudes via ingroup identification did not reach significance (*b* = 1.81, *CI*_*95%*_ = 0.22, 4.59, *p* = .064). However, in line with our predictions, the effect of identification on outgroup attitudes was moderated by SDO (*b* = -4.96, *CI*_*95%*_ = -11.00, 0.68, *p* < .07) resulting in a significant conditional indirect effect for High-SDOs (+1 *SD* above the mean; *b* = 2.75, *CI*_*95%*_ = 0.25, 7.18, *p* = .076) and a non-significant conditional indirect effect for Low-SDOs (-1 *SD* below the mean; *b* = 0.86, *CI*_*95%*_ = -0.15, 2.72, *p* = .22).

Please note that for a number of effects in Study 3, the *p*-value indicated a non-significant effect (i.e., *p* > .05) while the bootstrapping confidence intervals pointed towards significance, as evidenced by the confidence intervals excluding zero. A growing body of research is placing heavier emphasis on using bootstrapping techniques in assessing model parameters as they are better able to handle skewed data, retain the most power in small samples, and return accurate Type I error rates [58,59]. It is for this reason that we rely on the bootstrap estimates in gauging statistical significance.

#### Moderated mediation analyses for Catholics

The direct effect of cross-group friendship on positive outgroup attitudes (without inclusion of identification) was significant (*b* = 6.87, *CI*_*95%*_ = 2.64, 11.32, *p* = .001).

In the full moderated mediation model, a significant direct relationship between cross-group friendship and positive outgroup attitudes emerged (*b* = 7.03, *CI*_*95%*_ = 2.46, 11.29, *p* = .001). Also, cross-group friendship was negatively associated with ingroup identification (*b* = -.32, *CI*_*95%*_ = -0.54, -0.08, *p* = .005). However, ingroup identification was unrelated to positive outgroup attitudes (*b* = 1.33, *CI*_*95%*_ = -5.53, 7.11, *p* = .64) while SDO was negatively associated with positive outgroup attitudes (*b* = -9.38, *CI*_*95%*_ = -17.61, -1.36, *p* = .01).

The indirect effect of cross-group friendship on outgroup attitudes via ingroup identification did not reach significance (*b* = -.43, *CI*_*95%*_ = -2.71, 1.75, *p* = .64). The effect of identification on outgroup was not moderated by SDO for Catholics (*b* = -4.54, *CI*_*95%*_ = -11.49, 2.36, *p* = .13), making it meaningless to probe for conditional indirect effects at low and high levels of SDO for this subsample.

Please note that SDO did not moderate the direct relationship between cross-group friendship and outgroup attitudes for Catholic respondents (*p* = .13) in Study 3. SDO did, however, moderate the direct path from cross-group friendship to outgroup attitudes for Protestant respondents (*p* = .003). The relationship between cross-group friendship and outgroup attitudes approached significance for respondents high in SDO (+1 *SD*; *b* = 5.86, *CI*_*95%*_ = -0.64, 11.16, *p* = .036) while it was non-significant for respondents low in SDO (-1 *SD*; *b* = -4.17, *CI*_*95%*_ = -9.91, 2.56, *p* = .12). We also tested an alternative model in which outgroup attitudes are predicted by cross-group friendship, SDO, and identification as well as the interactions between these variables (including the three-way interaction). For the Protestant sample, cross-group friendship was not related to outgroup attitudes (*p* = .93) whereas ingroup identification and SDO were negatively and significantly related to outgroup attitudes (both *ps* ≤ .001). The two-way interaction between SDO and ingroup identification remained closed to significance (*p* = .07). SDO also interacted with cross-group friendships (*p* < .05, +1 *SD* SDO: *b* = 4.06, *p* = .21, -1 *SD* SDO: *b* = -3.65, *p* = .19). No other interactions were significant. This model yielded no significant interactions for the Catholic sample whereas positive contact was positively and significantly related to outgroup attitudes (*p* = .006), ingroup identification was not related to outgroup attitudes (*p* = .71), and SDO was negatively and significantly related to outgroup attitudes (*p* = < .001).

Lastly, we ran a modified multigroup analysis to check if the interaction terms were significantly different between the Catholic and Protestant samples. We compared the model where all structural paths were freely estimated between the two samples to one where we only constrained the interaction terms to equality. We compared the chi-square statistic, using the scaled chi-square test [60], for evidence of model invariance, which revealed that the two models were statistically equivalent, Δ*χ*^*2*^ = 0.01, *df* = 1, *p* = .91. Thus we did not find evidence of a significant difference between the two interaction terms for the two groups in the multigroup comparison. It is important to keep in mind though that the interaction term was of larger magnitude for, and statistically significant only in the case of, Protestants, not Catholics.

In sum, the results for Protestants (considered, historically, to be the high-status group) were similar to those observed in Studies 1 and 2: the association between reduced ingroup identification and outgroup attitudes was moderated by SDO. However, the indirect effect of cross-group friendship on outgroup attitudes via reduced identification emerged only among high-SDO Protestant respondents. Conversely, SDO did not moderate the relation between reduced ingroup identification and outgroup attitudes among Catholics, typically considered the low-status group in this context. We thus did not observe an indirect ingroup distancing effect for the Catholic respondents, although we did observe a positive direct effect of cross-group friendship on outgroup attitudes for this group. However, the multigroup analysis indicated no difference in the magnitude of the interaction effect between Protestants and Catholics. In sum, these analyses provide preliminary support for our hypothesis that contact affects outgroup attitudes via ingroup distancing, but only among high-SDO individuals. With regard to the role of group status, though, our results are more mixed. Studies 1 and 2 only replicated for majority/high-status group members. However, while SDO only significantly moderated the effect of identification on attitudes for the majority Protestant group, but not the Catholic minority group, the interaction terms themselves did not differ statistically. Given the small sample sizes for the two groups in this study we aimed to replicate our results in a larger sample in the same context in Study 4.

## Study 4

Similar to Study 3, Study 4 was conducted in the context of intergroup relations in Northern Ireland. However, predictions are tested using a community instead of a student sample.

### Method

#### Participants and Procedure

Respondents were 1,948 adults (978 Protestants (391 males, 587 females); 970 Catholics (353 males, 617 females); *M*_*age*_ = 45 years, age range: 18–92). Respondents were randomly sampled from six different towns in Northern Ireland and data were collected between March and October 2007 by a professional survey company (using computer aided personal interviewing techniques in respondents’ own homes). We followed the strict ethics code of the survey research company that collected the data (name of research company: Customer & Marketing Surveys Ltd.). Therefore no formal ethics waiver or approval was requested. Participants were assured that participation is voluntary, can be cancelled at any time and that data were anonymized. Informed consent was provided orally since that was the only practical means of doing so without compromising people's anonymity. Participants’ verbal consent was documented by the research company. The research company did not collect any identifying information such as name, address or telephone number from participants. Phone numbers were created by a computer program and were not visible to interviewers. Participants’ names were not recorded. Therefore data were anonymous when handed over to researchers. The data file is available in the Open Science Framework: https://osf.io/5hkm4/.

Respondents completed a larger battery of items, of which we use only a subsample in the present paper (see e.g., [61], which used other constructs from this dataset).

#### Measures

*Cross-group friendship* was measured using two items (‘How many of your close friends are OUTGROUPERS?’ (1 *= none*, 7 *= all*), ‘How often do you visit your close OUTGROUPER friends in their home?’ (1 *= never*, 7 *= very often*). The items were positively correlated (*r* = .40, *p* < .001) and treated as a combined index of cross-group friendship. *Ingroup identification* was measured using four items (e.g., ‘Being <INGROUP> is an important part of who I am’, ‘I identify with other <INGROUP>‘; 1 *= strongly disagree*, 5 *= strongly agree*). The four items formed a reliable scale (*α* = .91). *Outgroup attitudes* were measured using the same feeling thermometer used in Study 3. SDO was measured using eight items selected from Pratto et al.’s [24] SDO scale (e.g., ‘Inferior groups should stay in their place’, ‘Some groups of people are just more worthy than others’; 1 = *strongly disagree* to 5 = *strongly agree*; α = .60).

### Results and Discussion

#### Preliminary analyses

We carried out a series ANOVAs to test for group differences between Protestants and Catholics on all variables. Results showed no differences between Protestants and Catholics respondents on any of the variables included in the analyses in this sample (all *F*s (1, 1943) ≤ 3.44, all *p*s *>* .08, all *η*^*2*^ < .05). Again, testing for structural invariance revealed that the model was structurally non-invariant for the two subpopulations on our key hypothesized relationships, *Δχ*^*2*^ = 598.25, *df* = 13, *p* < .001, hence we report results separately for Protestants and Catholics.

We used the same analytic approach as in the previous studies. Table 4 reports means, standard deviations, and correlations of all variables.

**Table 4:** Means, standard deviations, and correlations for variables in Study 4 for Catholics (above the diagonal) and Protestants (below the diagonal).

Variable	Cross-group friendship	Ingroup identification	Pos. outgroup attitudes	SDO	*M*_Catholics_	*SD*_Catholics_
Cross-group friendship	–	-.12***	.17***	.04	2.90	1.43
Ingroup identification	-.23***	–	.03	.07*	3.78	.98
Positive outgroup attitudes	.42***	-.31***	–	.02	73.75	19.48
SDO	-.13***	.16***	-.21***	–	1.96	0.52
*M*_Protestants_	2.78	3.69	65.53	2.07	-	-
*SD*_Protestants_	1.39	1.00	20.00	.57	-	-

Note: SDO = Social Dominance Orientation.

*p < .05

***p < .001.

#### Moderated mediation analyses for Protestants

The direct effect of cross-group friendship on positive outgroup attitudes (without inclusion of identification) was significant (*b* = 0.71, *CI*_*95%*_ = 0.02, 4.88, *p* = .001).

In the full moderated mediation model, no significant direct relationship between cross-group friendship with the ethno-religious outgroup and positive outgroup attitudes emerged (*b* = 0.42, *CI*_*95%*_ = -0.16, 3.97, *p* = .036). Cross-group friendship was, however, negatively associated with ingroup identification (*b* = –.06, *CI*_*95%*_ = –0.16, –0.04, *p* < .001), and identification was associated with less positive attitudes (*b* = –5.29, *CI*_*95%*_ = –6.54, –3.81, *p* < .001), as was higher SDO (*b* = –3.33, *CI*_*95%*_ = *–*4.57, –1.93, *p* < .001).

The indirect effect of cross-group friendship on outgroup attitudes via ingroup identification was significant (*b* = 0.32, *CI*_*95%*_ = 0.16, 0.70, *p* < .001). Additionally and in line with our predictions, the effect of identification on outgroup attitudes was moderated by SDO (*b* = –1.79, *CI*_*95%*_ = –3.04, –0.51, *p* = .002) resulting in a significant conditional indirect effect for High-SDOs (+1 *SD* above the mean; *b* = 0.43, *CI*_*95%*_ = 0.25, 0.94, *p* < .001) as well as for Low-SDOs (-1 *SD* below the mean; *b* = 0.21, *CI*_*95%*_ = 0.10, 0.48, *p* = .001), yet the indirect relationship was stronger for individuals high in SDO.

#### Moderated mediation analyses for Catholics

The direct effect of cross-group friendship on positive outgroup attitudes (without inclusion of identification) was significant (*b* = 2.29, *CI*_*95%*_ = 1.41, 3.16, *p* < .001).

In the full moderated mediation model, the direct relationship between cross-group friendship and positive outgroup attitudes remained significant (*b* = 2.33, *CI*_*95%*_ = 1.44, 3.21, *p* < .001). Also, cross-group friendship was negatively associated with ingroup identification (*b* = –.09, *CI*_*95%*_ = –0.13, –0.04, *p* < .001). However, ingroup identification was unrelated to outgroup attitudes (*b* = .93, *CI*_*95%*_ = –0.32, 2.19, *p* = .14), as was SDO (*b* = .31, *CI*_*95%*_ = -0.94, 1.64, *p* = .62).

The indirect effect of cross-group friendship on outgroup attitudes via ingroup identification did not reach significance (*b* = -.08, *CI*_*95%*_ = -0.22, 0.03, *p* = .17). The interaction between identification and SDO also failed to reach significance (*b* = –.77, *CI*_*95%*_ = –2.10, 0.54, *p* = .22), making it meaningless to probe for conditional indirect effects at low and high levels of SDO for this subsample.

Please note that SDO moderated the direct effect between cross-group friendship and outgroup attitudes for Protestant respondents (*p* < .001) in Study 4. The relationship between cross-group friendships and outgroup attitudes was significantly stronger for individuals high in SDO (+1 *SD*; *b* = 5.47, *CI*_*95%*_ = 3.97, 7.59, *p* < .001) than it was for those low in SDO (-1 *SD*; *b* = 1.77, *CI*_*95%*_ = 0.85, 3.22, *p* < .001). The SDO by cross-group friendship moderation was not significant for Catholic respondents (*p* = .81). We also tested an alternative model, in which outgroup attitudes were regressed onto cross-group friendships, ingroup identity, SDO, and their various interactions. For the Catholic participants, cross-group friendships were positively associated with outgroup attitudes (*p* < .001) whereas neither SDO nor ingroup identity were related to outgroup attitudes (both *ps* ≥ .20). Moreover, none of the two-way interactions reached significance (*p* > .17). There was, however, a significant three-way interaction (*p* = .005). We decomposed the interaction looking at the relationship between ingroup identification and outgroup attitudes at the various combinations of high and low SDO and cross-group friendships. The following pattern of results emerged. For individuals who reported having relatively more cross-group friendships (+1 *SD*) and higher levels of SDO (+1 *SD*), the relationship between ingroup identification and outgroup attitudes was positive, but not significant (*b* = 1.49, *CI*_*95%*_ = -0.38, 3.39, *p* = .14). For individuals with fewer cross-group friendships (-1 *SD*) and high levels of SDO (+1 *SD*), the relationship between ingroup identification and outgroup attitudes followed an ethnocentric pattern, but was non-significant (*b* = -1.61, *CI*_*95%*_ = -3.92, 0.81, *p* = .14). For individuals with relatively more cross-group friendships and low levels of SDO, there was no relationship between ingroup identification and outgroup attitudes (*b* = .09, *CI*_*95%*_ = -2.00, 2.20, *p* = .93) whereas for individuals with fewer cross-group friendships and low SDO, ingroup identification was positively related to outgroup attitudes (*b* = 3.19, *CI*_*95%*_ = 0.98, 5.45, *p* = .002). For the Protestant sample, cross-group friendships were directly associated with outgroup attitudes (*p* < .001) and ingroup identification and SDO were negatively associated with outgroup attitudes (both *ps* < .001). Moreover, there was evidence of a two-way interaction between SDO and cross-group friendships (*p* < .001; +1 *SD* in SDO: *b* = 6.09, *CI*_95_% = 4.60, 7.68, *p* < .001, -1 *SD* in SDO: *b* = 1.14, *CI*_95%_ = 0.24, 4.44, *p* < .001) as well as an interaction between ingroup identification and cross-group friendships (*p* = .002; +1 *SD* in friendship: *b* = -1.01, *CI*_95%_ = -3.94, 2.47, *p* = .44, -1 *SD* in friendship: *b* = -8.02, *CI*_95%_ = -11.41, -4.96, *p* < .001). There was, however, no interaction between SDO and ingroup identification (*p* = .51) and no three-way interaction (*p* = .42).

As in Study 3, we ran a multigroup analysis to test specifically if the interaction terms were significantly different between the two samples, which indicated that the two interaction terms were statistically equivalent (Δ*χ*^*2*^ = 1.39, *df* = 1, *p* = .24).

Results for Study 4 were similar to Studies 1–3, such that the relationship between identification and outgroup attitudes was moderated by SDO, but only for the higher-status Protestant respondents. However, contrary to Study 3, the overall indirect effect of cross-group friendship on outgroup attitudes via reduced ingroup identification was also significant for this group; the effect was, however, stronger for High-SDOs than for Low-SDOs. For Catholics, no indirect effect via ingroup distancing occurred, yet similar to Study 3, the direct effect of cross-group friendship on outgroup attitudes was significant. Again, SDO did not moderate the relationship between ingroup identification and outgroup attitudes in the low status Catholic subsample. However, as in Study 3, the multigroup analysis did not indicate a significant difference in the moderating effect of SDO between subsamples leading to an ambiguous pattern of results.

The fact that we witnessed no differences in the moderating role of SDO between Protestants and Catholics in Studies 3 and 4 –as well as a significant, albeit weaker, indirect effect for low-SDO Protestants in Study 4 –might be due to unique particularities of the Northern Ireland conflict, especially the aforementioned potential complexities surrounding current status relations. Shifts in numerical proportions and precarious status relations may motivate Protestants to attempt to retain their majority status and Catholics to assert their position in the status hierarchy. It is, perhaps, for this reason that identification was associated with outgroup attitudes even among low-SDO individuals in the Protestant group in Study 4. Unclear status-relations could also have caused the lack of significant differences in the moderating role of SDO between both groups in Studies 3 and 4. Study 5 therefore sought to replicate the hypothesized effects in a different context of intergroup relations, involving more clearly defined status-hierarchies.

## Study 5

Study 5 examined the hypothesized relationships in the context of intergroup relations between White British majority and Asian British minority (of Muslim religion, with either Bangladeshi or Pakistani heritage) students in an ethnically mixed high school. At the time of data collection, approximately 59% of the enrolled students were of White British background and 34% were of Asian British background. The school is located in a town in the North of England, UK, which had witnessed extreme interethnic tensions approximately 10 years prior to data collection.

### Method

#### Participants and Procedure

Respondents were 594 adolescent (361 White British (156 males, 205 females); 233 Asian British 99 males, 134 females); *M*_*age*_ = 16.44; age range: 16–18 years) at an ethnically mixed sixth-form college (high school) in the North of England. Data were collected between October and November 2010, using a web-based survey, administered to students during regular class hours. The questionnaire was approved by Oxford University's Central University Research Ethics Committee (CUREC). Informed consent was provided in written format, by students themselves. In addition, parental consent was obtained. The data file is available in the Open Science Framework: https://osf.io/5hkm4/.

#### Measures

Among a number of measures unrelated to the present study, students completed measures of cross-group friendship (with ethnic outgroup members), ingroup identification (with the ethnic ingroup), outgroup attitudes (towards the ethnic outgroup) and SDO. *Cross-group friendship* was measured using five items (‘How many of your friends at <COLLEGE NAME> are <OUTGROUP>?’ (0 *= none*, 7 *= all*), ‘How often do you spend time with these friends outside of college (e.g., in town, in your home, or elsewhere)?’ (0 = never, 7 = very often), ‘How many of your friends from outside of <COLLEGE NAME> are <OUTGROUP>?’ (0 *= none*, 7 *= all*), ‘And in total numbers, how many <OUTGROUP> friends would you say you have?’ (0 *= 0*, 7 *= 11 or more*), ‘In general, how much do you enjoy spending time with your <OUTGROUP> friends (whether they are from college or outside of college)?’ (1 *= not at all*, 7 *= very much*)). The five items formed a reliable scale (*α* = .77). *Ingroup identification* was measured using four items (‘I feel good about being <INGROUP>‘, ‘I feel close to other <INGROUP>‘, ‘Being <INGROUP> is an important part of who I am’, ‘I am a typical <INGROUP>‘; 1 *= strongly disagree*, 7 *= strongly agree*). The four items formed a reliable scale (*α* = .88). *Outgroup attitudes* were measured using two items (‘How much do you like <OUTGROUP>?’, ‘How positive do you feel about <OUTGROUP>?’; 1 *= not at all*, 7 *= very*). The items were highly correlated (*r* = .89, *p* < .001) and treated as a combined index. SDO was measured using a short, four item-version of the SDO scale (see also Sidanius et al., 2008): ‘Inferior groups should stay in their place’, ‘It’s probably a good thing that certain groups are at the top and others at the bottom’, ‘We should do what we can to equalize conditions for different groups (R)’, ‘We should increase social equality (R)’ (1 *= strongly disagree*, 7 *= strongly agree*). The four items (with items marked R reverse scored) formed a reliable scale (*α* = .79).

### Results and Discussion

#### Preliminary analyses

A series of ANOVAs revealed significant differences in cross-group friendship, (*F*(1, 553) = 89.11, *p* < .001, *η*^*2*^ = .139), ingroup identification (*F*(1, 543) = 6.72, *p* = .01, *η*^*2*^ = .011), outgroup attitudes (*F*(1, 509) = 43.38, *p* < .001, *η*^*2*^ = .079), and SDO (*F*(1, 466) = 8.77, *p* < .01, *η*^*2*^ = .018) between the White British majority and the Asian British minority students.

Next (given that we had multiple items for these measures in this study, thus permitting this analysis in this study) we sought to confirm that the five items hypothesized to measure cross-group friendships and the two items hypothesized to tap outgroup attitudes were valid indicators of their respective constructs. To accomplish this, we entered the five contact items and two outgroup attitude items into confirmatory factor analysis (CFA; maximum likelihood estimator with robust standard errors). We ran two CFAs: The first CFA specfied the seven items as loading onto their hypothesised constructs; the second CFA constrained all seven items to load onto a single, *general contact* factor. The model specifying separate cross-group friendship and outgroup attitude factors showed acceptable model fit, χ^2^ (13) = 91.635, *p* < .001, χ^2^ /*df* = 7.04, CFI = .94, RMSEA = .10 [.09, .13], SRMR = .07, whereas the factor model in which all cross-group friendship and outgroup attitude items loaded onto a single factor showed poor model fit, χ^2^ (14) = 378.746, *p* < .001, χ^2^ /*df* = 27.05, CFI = .71, RMSEA = .22 [.20, .24], SRMR = .14 (Δ*χ*^*2*^ = 286.27, *df* = 1, *p* < .001). These results support the interpretation that cross-group friendships and outgroup attitudes are distinct factors, and are consistent with previous research confirming the factor-analytic validity of cross-group friendships and outgroup attitudes ([62–65] for a review of the psychmoteric properties of the most frequently used variables in intergroup contact research).

Similar to Studies 3 and 4, we first tested for structural invariance of our model across the White British majority and the Asian British minority sample, which revealed that the model was structurally non-invariant for the two groups (*Δχ*^*2*^ = 54.60, *df* = 13, *p* < .001). We therefore report analyses separately for the two groups. We used the same analytic approach as in the previous studies. Table 5 reports means, standard deviations, and correlations of all variables.

**Table 5:** Means, standard deviations, and correlations for variables in Study 5 for Asian British (above the diagonal) and White British (below the diagonal).

Variable	Cross-group friendship	Ingroup identification	Pos. outgroup attitudes	SDO	*M*_AsianBritish_	*SD*_AsianBritish_
Cross-group friendship	–	-.10	.50***	-.14	3.68	1.19
Ingroup identification	-.17**	–	-.13	-.22**	5.44	1.54
Positive outgroup attitudes	-44***	-.25***	–	-.15*	5.32	1.29
SDO	-.23***	.26***	-.52***	–	2.56	1.29
*M*_WhiteBritish_	2.79	5.22	4.54	2.93	-	-
*SD*_WhiteBritish_	1.03	1.31	1.31	1.34	-	-

Note: SDO = Social Dominance Orientation.

*p < .05

**p < .01

***p < .001

#### Moderated mediation analyses for White British sample

The direct effect of cross-group friendship on positive outgroup attitudes (without inclusion of identification) was significant (*b* = .56, *CI*_*95%*_ = .42, .70, *p* < .001).

In the full moderated mediation model, the direct relationship between cross-group friendship and positive outgroup attitudes remained significant (*b* = .40, *CI*_*95%*_ = .28, .53, *p* < .001). Also, cross-group friendship was negatively associated with ingroup identification (*b* = –.16, *CI*_*95%*_ = –.25,–.06, *p* = .001). However, ingroup identification was unrelated to outgroup attitudes (*b* = –.12, *CI*_*95%*_ = –.26, .01, *p* = .079). SDO was negatively associated with positive outgroup attitudes (*b* = –.54, *CI*_*95%*_ = -.66, -.42, *p* < .001).

The indirect effect of cross-group friendship on outgroup attitudes via ingroup identification did not reach significance (*b* = .02, *CI*_*95%*_ = .00, .05, *p* = .151). However, the effect of identification on outgroup attitudes was moderated by SDO (*b* = –.14, *CI*_*95%*_ = –.26,–.01, *p* = .024) resulting in a significant conditional indirect effect for High-SDOs (+1 *SD* above the mean; *b* = .04, *CI*_*95%*_ = .01, .08, *p* = .035) and a non-significant conditional indirect effect for Low-SDOs (-1 *SD* below the mean; *b* = –.003, *CI*_*95%*_ = –.03, .03, *p* = .828).

#### Moderated mediation analyses for Asian British sample

The direct effect of cross-group friendship on positive outgroup attitudes (without inclusion of identification) was significant (*b* = .55, *CI*_*95%*_ = .40, .69, *p* < .001).

In the full moderated mediation model, the direct relationship between cross-group friendship and positive outgroup attitudes remained significant (*b* = .52, *CI*_*95%*_ = .38, .66, *p* < .001). However, cross-group friendship was unrelated to ingroup identification (*b* = -.09, *CI*_*95%*_ = –.22, .04, *p* = .16) and identification also failed to predict outgroup attitudes (*b* = -.10, *CI*_*95%*_ = –.24, .06, *p* = .19), as did SDO (*b* = -.13, *CI*_*95%*_ = -.31, .06, *p* = .18).

Thus, the indirect effect of cross-group friendship on outgroup attitudes via ingroup identification did not reach significance (*b* = .01, *CI*_*95%*_ = -.10, .03, *p* = .34). Moreover, the effect of identification on outgroup attitude was not moderated by SDO for the Asian-British sample (*b* = -.14, *CI*_*95%*_ = -.30, .02, *p* = .07), making probing for conditional indirect effects at low and high levels of SDO meaningless for this subsample. In sum, we were unable to confirm our hypotheses among the minority sample, and witnessed only a significant direct effect of cross-group friendship on attitudes.

SDO did not moderate the direct effect between cross-group friendship and outgroup attitudes, for either Asian British (*p* = .21) or White British (*p* = .38) students in Study 5. For the White British sample, cross-group friendships were associated with more favourable attitudes towards the outgroup (*p* < .001) whereas ingroup identification (*p* = .024) and SDO (*p* < .001) were associated with less favourable attitudes towards the outgroup. Moreover, none of the two-way interaction terms reached significance (*p* ≥ .29). We found, however, a significant three-way interaction between cross-group friendships, ingroup identification, and SDO (*p* = .016). Simple slopes analyses revealed that, for individuals with more cross-group friendships (+1 *SD*) who were also high in SDO (+1 *SD*), ingroup identification was negatively, though non-significantly related to outgroup attitudes (*b* = -.10, *CI*_*95%*_ = -0.39, 0.17, *p* = .52), whereas for individuals high in SDO (+1 *SD*) with relatively few cross-group friendships (-1 *SD*), ingroup identification was related negatively to outgroup attitudes (*b* = -.41, *CI*_*95%*_ = -0.62, -0.21, *p* < .001). For individuals low in SDO (-1 *SD*) with relatively more cross-group friendships (+1 *SD*), ingroup identification was negatively related to outgroup attitudes (*b* = 25, *CI*_*95%*_ = -0.46, -0.004, *p* = .039). Individuals low in SDO (-1 *SD*) and with few cross-group friendships (-1 *SD*) showed no association between ingroup identification and outgroup attitude (*b* = .06, *CI*_*95%*_ = -0.15, 0.25, *p* = .60). For the Asian sample, cross-group friendships was positively and significantly associated with outgroup attitudes (*p* < .001), whereas neither ingroup identity nor SDO was significantly associatied with outgroup attitudes (both *ps* ≥ .15). Moreover, two interaction terms reached statistical significance, namely the interaction between SDO and cross-group friendships (*b* = .15, *CI*_*95%*_ = 0.007, 0.30, *p* = .044; +1 *SD* in SDO: *b* = .63, *CI*_*95%*_ = 0.43, 0.84, *p* < .001, -1 *SD* in SDO: *b* = .29, *CI*_*95%*_ = 0.06, *p* = .022), and the interaction between ingroup identification and cross-group friendships (*b* = .23, *CI*_*95%*_ = 0.09, 0.38, *p* = .002; +1 *SD* cross-group friends: *b* = 0.23, *CI*_*95%*_ = 0.04, 045, *p* = .061, -1 *SD* cross-group friends: *b* = -0.31, *CI*_*95%*_ = -0.53, -0.05, *p* = .007).

We ran a multigroup analysis similar to that in Study 3 to test if the interaction terms were significantly different between the two samples. The results indicated that the two interaction terms were statistically equivalent, Δ*χ*^*2*^ = 0, *df* = 1, *p* = 1.00.

In sum, the results of Study 5 for the high-status White British majority sample corresponded with the results of Study 1. We found a significant indirect effect of cross-group friendship on outgroup attitudes via reduced ingroup identification for High-SDOs only. We also observed a significant direct effect of cross-group friendship on outgroup attitudes, such that the conditional indirect effect reflected a partial mediation effect. However, similar to Studies 3 and 4, we did not observe the moderated mediation effect in the low-status Asian British sample, but only observed a significant direct, positive effect of cross-group friendship on outgroup attitudes. However, again in Study 5 multigroup analysis provided no evidence to support the claim that the moderating role of SDO on the relationship between ingroup identification and outgroup attitudes was reliably different between the White majority and the Asian minority group.

In a final step, we sought to aggregate our findings across studies. In the next section we therefore present evidence from a meta-analytical comparison of effects in Studies 1–5.

## Meta-analytical Summary of Results

To further test our predictions, we conducted a meta-analysis across the five studies reported in the present paper. We focused on the moderating effect of SDO on the relationship between ingroup identification and outgroup attitudes and calculated the mean moderating effects for majority (Studies 1–5) and minority (Studies 3–5) group members. We utilized the Meta-F macro [66] testing effects under the assumptions of the meta-analytic random effects model [67]. Standardized regression effects (i.e., *β*s) were inverse variance weighted [68].

The mean effect was *β* = -.083 (*CI*_*95%*_ = -.12, -.05, *p* < .001) for the majority samples and *β* = -.062 (*CI*_*95%*_ = -.12, -.01, *p* = .03) for the minority samples. Notably, the mean effect for the minority samples was significant (albeit weaker than in the majority samples) while the single effects in Studies 3–5 failed to reach significance. The difference between the mean effects was not significant (Cochran’s *Q* between groups = .43, *df* = 1, *p* = .51). However, this result should be interpreted with caution because this test is clearly underpowered. Cochran’s Q as a test of heterogeneity between groups is known to suffer from low power when sample size (i.e., number of studies) is small [69].

## General Discussion

In five studies, we tested an ingroup distancing effect of cross-group friendship on outgroup attitudes; i.e., we investigated whether cross-group friendship was associated with more positive outgroup attitudes via ingroup reappraisal, operationalized here as (reduced) ingroup identification. Responding to mixed prior empirical support concerning the relation between ingroup identification and outgroup attitudes, we hypothesized that the indirect effect of cross-group friendships on outgroup attitudes via reduced ingroup identification is qualified by Social Dominance Orientation (SDO). In line with research on Social Dominance Theory [48], we showed, in five different samples obtained, that ingroup identification mediated the effect of cross-group friendship on outgroup attitudes only for high-SDO but not for low-SDO individuals. The respective pattern of results, a moderated mediation, was replicated in three different intergroup contexts. We also tested alternative models (e.g., an additional moderation of the effect of cross-group contact on outgroup attitudes by SDO, cf. [12,36]; and a model in which outgroup attitudes are predicted by cross-group friendship, SDO, and identification as well as the interactions between these variables). However, no consistent systematic results for these models occurred across studies while our moderated mediation model was replicated across all of the studies. However, the moderated mediation was only significant in subsamples consisting of members of majority groups (i.e. Germans without migration background in Germany, Protestants in Northern Ireland and White British in England). To put if differently, SDO functioned as a moderator in high-status groups only, and was not a moderator in low-status groups (i.e., Catholics in Northern Ireland and Asian British in England).

Can we conclude that the hypothesized moderated mediation effect of cross-group friendship is limited to high-status groups? Our research cannot provide a definite answer to this question. There is some evidence in favor of this prediction: Across the five studies, the moderating effect of SDO occurred only among members of high-status majority groups (Studies 1–5), but not members of low-status minority groups (Studies 3, 4, and 5). However, we need to keep in mind that the specific multigroup analyses testing for invariance of the interaction terms in Studies 3–5, did not indicate a significant difference of the interaction effect of SDO between high-status and low-status samples. Moreover, the meta-analytic results are consistent with this last: Although the single effects for the minority samples in Studies 3–5 failed to reach significance, the mean effect for minority samples in the meta-analysis was significant. This is likely due to the higher statistical power obtained through the meta-analytic aggregation that makes it easier to detect small effects. However, it is nonetheless important to note that on a descriptive level the effect for majority samples (*β* = -.083) was higher than for minority samples (*β* = -.062). It is thus not surprising that the meta-analytic moderation failed to reach significance due to power problems that are caused by a comparison of 5 vs. 3 studies.

We can only speculate why group status did not influence the moderating role of SDO in the multigroup comparisons of the interaction effect, despite there being clear differences in statistical significance and magnitude of the interaction effects between the majority and minority groups when the analyses were run separately for each group. One reason might be that we studied contexts with rather unclear status relations. As mentioned above the majority-minority hierarchy in Northern Ireland (Studies 3 and 4) is not as clear as it used to be [56]. Moreover, it remains arguable whether the Asian minority students in the college considered in Study 5, although the numerical minority, can clearly be considered as the low-status group. The proportion of Asian minority students in this particular college was 34%, which is considerably higher than the national average not only for this particular minority group but indeed for ethnic minority groups more generally (14%; [70]). It may thus be that this relatively large numerical proportion in the college has implications for group status boundaries, whereby status hierarchies may be less pronounced in this context than elsewhere.

Notwithstanding the somewhat mixed pattern of findings, we believe that our research make an important contribution to the literature on intergroup contact generally and on deprovincialization specifically. Most prior research concerning the deprovincialization hypothesis has examined ingroup distancing and reappraisal in the context of secondary transfer effects of contact [6,7,13]. This work has thus primarily considered the extent to which intergroup contact with a primary outgroup affects attitudes towards a secondary outgroup via reduced ingroup identification or ingroup attitude (some studies also considered primary outgroup attitudes as an additional/parallel mediator, see [6]). However, the deprovincialization hypothesis also applies more generally to intergroup contact effects, and indeed needs to be established in the context of primary outgroups too, as we have shown here. Pettigrew’s [1] initial definition of deprovincialization did not speak solely to explaining secondary outgroup attitudes, but argued that contact prompts a reappraisal of one’s ingroup, which can bring about positive effects for intergroup relations more generally. In other words, the ingroup distancing effect is not only of relevance for explaining secondary transfer effects of contact, but also for explaining intergroup contact effects on (primary) outgroup attitudes–contact with a target outgroup can thus also affect attitudes towards the same target outgroup, via reduced identification.

Moreover, as we have shown here, such effects occur only for certain individuals; that is for (high-status) individuals high in Social Dominance Orientation. We have thus demonstrated that SDO moderates the indirect effect of cross-group friendship on outgroup attitudes via distancing from the ingroup, in this case via reduced ingroup identification. So far evidence concerning such effects of ingroup reappraisal has been mixed, with some studies supporting [5], and others failing to present evidence [6,13] for the mediating role of ingroup distancing. The moderating role of SDO for the relationship between ingroup distancing and outgroup distancing might explain the ambiguity of prior results. Our studies show a moderated mediation effect of cross-group friendship on outgroup attitudes, to the extent that individuals who generally endorse hierarchical relations between groups showed a more pronounced effect of ingroup identification on outgroup attitudes, and thus a stronger indirect effect of cross-group friendship on outgroup attitudes. No prior research has previously considered such conditional indirect effects. One could speculate that due to ingroup distancing the instrumentality of prejudice declines for individuals high in SDO because for these individuals it is less important to maintain ingroup superiority in relation to an outgroup with whom a cross-group friendship exists. In other words, for high-SDO individuals dis-identification following cross-group friendship results in a weakened ingroup-focus in SDO [32].

In addition, a mediating effect of reduced ingroup identification occurred for high-status groups but not for low-status groups. Although cross-group friendship in Studies 3 and 4 (but not in Study 5) was associated with reduced ingroup identification, in neither of the two studies was identification associated with outgroup attitudes, regardless of level of SDO, among the minority groups. This might be due to the different meaning of identification for high-status and low-status groups [43,71]. Moreover, minority groups often tend to identify more strongly with their ingroup than majority groups [72], an effect we also observed here, and minority groups may thus be less likely to dis-identify with their ingroup in response to contact, as we observed in Study 5. Indeed, it may not even be advantageous to reduce ingroup identification among minority groups, because identification, especially for minority groups, can serve important adaptive functions. For example, identification is often associated with better psychological health outcomes [73], and can protect from negative identity-related stressors, such as discrimination [74] or perceived threat [75].

It is, however, important to keep in mind that we observed consistent direct effects between cross-group friendship and outgroup attitudes among members of the minority groups, whereas for the majority groups we primarily witnessed the conditional indirect effects (with the exception of Study 5, where cross-group friendship also exerted a direct effect on outgroup attitudes for the White British majority sample, in addition to the moderated mediation effect). This suggests that intergroup contact may work via different processes for majority and minority members.

Notwithstanding the contributions of our work, we acknowledge a number of methodological and conceptual limitations of our studies. Most importantly, because much of our reasoning is based on Pettigrew’s [1] deprovincialization hypothesis, it should be kept in mind that ingroup distancing in the form of reduced ingroup identification is unlikely to be the only process of deprovincialization. In our studies we sought to make use of, by and large, comparable measures of ingroup identification as operationalizations of deprovincialization, in order to respond to the previously mixed evidence base concerning the link between identification and outgroup attitudes. However, future research should seek to replicate our findings using other plausible measures of deprovincialization, such as ingroup re-evaluation or ingroup attitudes [1]. Also, endorsement of multiculturalism could be related to deprovincialization [7,76], because endorsement of multiculturalism has been shown to lead to reduced prejudice towards outgroups [15]. Closely related to this idea, valuing diversity–an important part of multiculturalism–has been shown to reduce discrimination of outgroups [77].

One might also consider extensions of the deprovincialization hypothesis, to consider multiple identity processes as a mediator in the relationship between contact and outgroup attitudes. One such process is social identity complexity [78], which reflects variations in the extent to which individuals perceive their multiple ingroups to be interrelated or differentiated [13]. Future research should therefore not only study whether ingroup identification decreases as a result of contact but also whether the general pattern of (multiple) self-categorization and identification changes to the extent that individuals start developing a more inclusive sense of self across their multiple identities, which may have positive effects for intergroup relations. Especially for low-status minority groups it is possible that cross-group contact does not reduce ethnic identification but may increase identification with the broader national category [46]. Future research should thus test not only subgroup identification (as we have done here), but also superordinate identification as a mediator of contact effects, and examine whether SDO also qualifies any potential effects of superordinate identity on attitudes, for both majority and minority groups.

Finally, one major limitation of our research is that we were unable to study directly the process of *intra*individual ingroup distancing but, rather, tested the *inter*individual relationship between contact and ingroup identification. In other words, although we found a relationship between cross-group contact, ingroup identification and outgroup attitudes over time in longitudinal Study 2, we cannot draw causal conclusions from these correlational data. Future studies should therefore try to replicate our findings in experimental settings. These limitations should, however, be set against the strengths of the present paper, which include the multiple studies, conducted across a range of settings, and with higher external validity associated with use of non-student samples in four of our five studies.

To conclude, we have shown across multiple studies and a range of intergroup contexts that ingroup distancing effects do not occur universally but are dependent on individuals’ SDO. In so doing, our research has opened up important avenues for future research that seeks to understand when, and how, intergroup contact becomes an effective means for improving intergroup relations.
